# Inhibin B and antiMüllerian hormone as surrogate markers of fertility in male and female Crohn’s disease patients: a case-control study

**DOI:** 10.3389/fmed.2024.1374603

**Published:** 2024-04-25

**Authors:** Ana Gutiérrez, Roser Muñoz-Pérez, Pedro Zapater, Cristina Mira, Andrés Rodríguez, Laura Sempere-Robles, María Eugenia Torregrosa, Rocio Alfayate, Violeta Moreno-Torres, Lorena Bernal, Olivia Belén-Galipienso, Jose Ignacio Cameo, Paula Sirera, Belen Herreros, Puri Bernabeu, Oscar Moreno-Pérez, Lucía Madero-Velázquez

**Affiliations:** ^1^Department of Gastroenterology, Hospital General Universitario Dr Balmis de Alicante, Alicante, Spain; ^2^Instituto de Instituto de Investigación Sanitaria y Biomédica de Alicante (ISABIAL), Alicante, Spain; ^3^Centro de Investigación Biomédica en Red en Enfermedades Hepáticas y Digestivas (CIBERehd), Madrid, Spain; ^4^Department of Clinical Pharmacology, Hospital General Universitario Dr Balmis de Alicante, Alicante, Spain; ^5^Universidad Miguel Hernández, Elche, Spain; ^6^Department of Gastroenterology, Hospital General Universitario Elche, Elche, Spain; ^7^Clinical Analysis Department, Hospital General Universitario Dr Balmis de Alicante, Alicante, Spain; ^8^Department of Gastroenterology, Hospital Marina Baixa, Villajoyosa, Spain; ^9^Department of Psychology, Hospital General Universitario Dr Balmis de Alicante, Alicante, Spain; ^10^Department of Endocrinology and Nutrition, Hospital General Universitario Dr Balmis de Alicante, Alicante, Spain

**Keywords:** fertility, Crohn’s disease, antiMüllerian hormone, ovarian reserve, follicle stimulating hormone (FSH), inhibin-B, Sertoli cell, sexual dysfunction

## Abstract

**Background:**

Several studies suggest that women with Crohn disease (CD) have reduced fertility due to decreased ovarian reserve, among other causes. On the other hand, male CD patients could have difficulties conceiving. The present study aimed to test the effect of CD on both male and female fertility potential, Sertoli cell function and ovarian reserve, assessed by inhibin-B (IB) plus IB:FSH ratio (IFR) and antiMüllerian hormone (AMH), respectively. Sexual dysfunction (SD) was studied as secondary endpoint.

**Methods:**

We performed a cross-sectional, case–control study. Serum IB levels plus IFR were measured in 58 men with CD and compared to 25 age-matched healthy controls (HC). Serum AMH levels were measured in 50 women with CD and in 30 HC matched by age. SD was assessed by means of the International Index of Erectile Function (IIFE-15) in males and the Index of Female Sexual Function (IFSF) in women.

**Results:**

A total of 108 CD patients and 55 HC were included. IB serum levels were significantly lower in CD men than in HC (177 ± 58 vs. 234 ± 75 pg./mL, *p* = 0.001). IFR was also decreased in CD patients compared to HC (58.27 ± 59.5 vs. 91.35 ± 60.04, *p* = 0.014). Women with CD > 30 years had lower serum AMH levels compared to HC (1.15 ± 0.74 *vs*. 2.14 ± 1.68 ng/mL, *p* = 0.033). In addition, CD women >30 years presented a serum AMH < 2 ng/mL more frequently than HC (90% *vs*. 40%, *p* = 0.004). The prevalence of SD was significantly higher among both male and female CD patients compared to HC, without association to fertility potential. Age was the only predictor of low ovarian reserve.

**Conclusion:**

Testicular Sertoli cell function assessed through serum IB levels and IFR is decreased in CD male patients compared to HC, regardless of age. Age > 30 years is the single independent predictor of reduced ovarian reserve in women with CD. These results should be confirmed in further studies in order to properly counsel patients with CD and desire for offspring.

## Introduction

Inflammatory Bowel Diseases (IBD), including both Crohn’s Disease (CD) and Ulcerative Colitis (UC), are chronic, progressive and disabling diseases characterized by a remitting and relapsing course. CD occurs early in the reproductive life of patients. Many CD patients are concerned about potential fertility problems. Previous data suggest reduced fertility among female CD patients, particularly in those with active disease (1, 2).

The reduction in fertility has been linked to inflammation affecting the Fallopian tubes, surgical procedures leading to adherences, or a premature decline in ovarian reserve (3). Furthermore, some studies also suggest that male IBD patients with active disease are more likely to report difficulty conceiving (4). The use of surrogate markers of fertility potential could be useful for counseling IBD patients (both female and male) with desire for offspring. Inhibin B (IB) is a dimeric glycoprotein synthesized in testicular Sertoli cells and in germ cells, and it is a marker of the functional status of the seminiferous epithelium, particularly the Sertoli cells (5). In adult men, serum IB levels are negatively correlated with serum follicle stimulating hormone (FSH) levels and positively correlated with sperm count, sperm concentration, Sertoli cell number and testicular volume, suggesting a paracrine role of IB in regulating spermatogenesis (6). IB levels are a more sensitive marker of male infertility than other available hormones, regardless of etiology, as they are a direct marker of Sertoli cell function and an indirect marker of spermatogenesis (7, 8). However, to date, very few data have been published regarding IB or IB:FSH ratio (IFR) in male patients with IBD.

On the other hand, ovarian reserve, which represents the number of primordial follicles, is estimated using serum antiMüllerian hormone (AMH) levels (5). AMH is a member of the transforming growth factor family and is produced in the ovary by granulosa cells of early developing follicles. There is a strong correlation between serum AMH levels and the number of developing follicles in the ovaries, which decreases with advancing age (9). AMH levels are not altered by use of hormonal contraception or parity and vary slightly during the menstrual cycle. Because of these facts, AMH could be a reliable marker of ovarian reserve and subsequently of fertility. Some previous studies have suggested that AMH is lower in women with IBD, but data are scarce and controversial (10–17). Therefore, IB plus IFR and AMH as fertility surrogate markers may be useful tools to study the fertility potential in both male and female patients with CD, in order to counsel properly those seeking to have children.

IBD could affect negatively sexual function, indeed the prevalence of sexual dysfunction (SD) has been estimated to be 40–60% among women with IBD and 15–25% in men, clearly higher than those reported in general population (18–21). Moreover, SD could be cause or consequence of subfertility. In fact the relationship between them is often bidirectional; for instance medical procedures to investigate and treat subfertility may increase anxiety and affect sexual functioning (22). Nevertheless studies regarding association between subfertility and SD report conflicting results (23, 24). Therefore, the aim of this study was to compare serum IB levels plus IFR between male CD patients and healthy controls (HC) and serum AMH levels between women with CD and HC, and to identify CD factors associated with impaired fertility and low ovarian reserve. In parallel we assessed SD, comparing it between CD patients and controls, and its potential association to impaired fertility.

## Materials and methods

### Study population/study design

This was a cross-sectional, observational, case–control study including consecutive CD outpatients, aged 18–40 years, and healthy controls (HC) matched by gender and age (ratio case/control: 2:1). The study protocol followed the Declaration of Helsinki and was approved by the Research Ethics Committee of the Hospital Universitario Dr. Balmis, Alicante, Spain (PI2019/093). All participants were fully informed and provided written informed consent to participate. A total of 163 subjects were enrolled from January 2020 to January 2022. Fifty reproductive-age women and 58 men, both of them diagnosed with CD disease, were included. The diagnosis of CD was established by endoscopic, radiological and histopathological features. In addition, 30 women and 25 age-matched men were included in the study as controls. HC were recruited from health care workers without a diagnosis of IBD.

Exclusion criteria for two groups included: age older than 40 years, menopause, pregnancy, lactation, ovarian disease or resection, previous diagnosis of malignancy or comorbid chronic disease, including hematologic or endrocine disease, previous exposure to cytotoxic drugs or pelvic radiotherapy, concomitant treatment with drugs that alter gonadal function (ganciclovir, ketoconazole, megestrol acetate, spironolactone and thiazide diuretics), opioid intake and psychiatric illness or cognitive deficit that makes it impossible to understand the study or regular follow-up.

### Sample size calculation

Assuming a mean (± standard deviation) AMH level of 2.20 ± 0.85 ng/mL in women of reproductive age with CD and 2.97 ± 1.20 ng/mL in healthy women according to previously published data (11) and a 2:1 case: control ratio, a total of 50 cases and 25 controls are needed to detect a difference of 0.77 ng/mL or greater, using a pooled standard deviation of 1.04 ng/mL, a two-side significant level α = 0.05 and a power (1-β) of 0.8. In the case of men with CD, there are no previous data on inhibin levels, so a similar number of cases and controls was estimated.

### Definitions

#### Clinical variables

IBD location and phenotype were defined according to the Montreal classification (25). CD activity was scored using the Harvey-Bradshaw index (HBI). CD patients with an HBI below 5 were considered to be in remission. Disease duration, extraintestinal manifestations, previous surgery and medical therapies, including steroids, immunosuppressants and biologics, were also collected from the digital medical records.

#### Demographical variables

Age, gender, smoking habit, alcohol consumption, body weight and height were measured in all subjects and body mass index (BMI) was calculated. Occupational status was categorized as student, employee, self-employed, unemployed, and retired. Marital status was classified as single, married, widowed, divorced and unmarried partner. Educational level was stratified as non-schooling, primary, secondary or higher education. Physical exercise was recorded and divided into 3 categories: sedentary lifestyle, <3 h per week or more than 3 h per week. Gravidity, parity and oral contraceptives use were also recorded.

### Sample collection

Morning venous blood samples were collected from the whole cohort. Serum samples from all patients were stored at −80°C until analysis. AMH and female hormone levels were measured during the early follicular phase (day 3 of the menstrual cycle) among women. Laboratory measurements included C-reactive protein (CRP), white blood cell count, albumin, hemoglobin, FSH, luteinizing hormone (LH), 25OH vitamin D, vitamin B12, estradiol, sex hormone binding globulin (SHBG), testosterone, AMH (also in men) and IB levels. Fecal calprotectin was measured in all CD patients. The concentration of free testosterone (FT) was calculated from the concentrations of total testosterone (TT), SHBG and albumin, using the equation described by Vermeulen et al. (26). Serum total testosterone (TT) (ng/mL; rr male 3–10 ng/mL), sex hormone binding globulin (SHBG) [nmol/L; rr 4–72 nmol/L, estradiol (pg/mL; rr female 30–100 pg./mL)], follicle stimulating hormone (FSH) [IU/L; reference range (rr) 1–8 IU/L] and luteinizing hormone (LH) (IU/L; rr 2–11.2 IU/L) were quantified by electrochemiluminescence immunoassay (ECLIA) using a Cobas 801 automated autoanalyzer (Roche Diagnostics, Mannheim, Germany). Albumin (g/dL; rr 3.5–5.2 g/dL) was determined by an immunoturbidimetric method, in a Cobas c 702 autoanalyzer (Roche Diagnostics, Mannheim, Germany).

### Anti-Müllerian hormone (ovarian reserve)

Serum AMH was measured by the electrochemiluminescence immunoassay ECLIA using a Cobas 801 automated autoanalyzer (Roche Diagnostics, Mannheim, Germany) with a limit of quantification 0.03 ng/mL and rr men 0.77–14.5 ng/mL. The assay precision was as follows: mean 0.91 ng/mL, coefficient of variance (CV) 1.5%; mean 5.76 ng/mL, CV 1.2%; mean 15.7 ng/mL, CV 1.5%.

### Inhibin-B (Sertoli cell function)

Serum IB levels were determined using an enzyme immunoassay (DSL-10-84100i Active©; DSL, Webster, TX, USA) with a sensitivity of 7 pg./mL. The intra-assay precision was as follows: mean 69 pg./mL, coefficient of variance (CV) 3.5%; mean 274 pg./mL, CV 4.6%; mean 472 pg./mL, CV 5.6%. Inter-assay precision (serum) was: mean 50.1 pg./mL, CV 7.6%; mean 188.4 pg./mL, CV 6.3%; mean 355.0 pg./mL, CV 6.2%.

### Inhibin-B: FSH ratio (Sertoli cell function)

Serum IB was determined by Gen II ELISA (Beckman Coulter INMUNOTECH, Prague, Czech Republic) with a limit of detection of 2.6 pg./mL and rr 80–350 pg./ mL. For each male subject, the IB: FSH ratio (IFR) was calculated as IB (pg/mL)/FSH (UI/L).

### Outcome variables and definitions

#### Primary

##### Low ovarian reserve

Ovarian reserve was assessed by AMH levels. AMH levels in women were evaluated according to age group, using the cut-offs of our local laboratory, considering low AMH level if AMH was <1.66 ng/mL up to 24 years, <1.18 ng/mL in 25–29 years, <0.67 ng/mL in 30–34 years, <0.77 ng/mL in 35–39 years, and < 0.01 ng/mL in 40–44 years. In addition, although there is ongoing debate about AMH cut-off levels in CD women, we considered low ovarian reserve AMH < 2 ng/mL in women under 40 years (11, 14). In an additional analysis, decreased ovarian reserve was defined by AMH < 1.1 ng/mL (27).

##### Impaired male fertility potential

We defined impaired male fertility potential as an IB level < 89 pg./mL or IFR < 23.5 (7).

#### Secondary

##### Sexual dysfunction and psychological functioning

SD was measured by the Index of Female Sexual Function (IFSF) in women (28). This index comprises six domains (sexual desire, arousal, lubrication, orgasm, satisfaction and pain), each one grading up to 6 points, leading to an overall score ranging from 2 to 36. Female SD was defined as an overall score < 26.55. Male SD was assessed using the International Index of Erectile Function (IIEF-15) (29). This index includes five domains (erectile function, orgasmic function, sexual desire, intercourse satisfaction and overall satisfaction). Male SD was defined as an IIFE score < 42.9. Values of the erectile function domain <26 were considered as erectile dysfunction. Psychological functioning was assessed using the Hospital Anxiety and Depression Scale [HADS]. The HADS-total consists of 14 items, divided into two 7-item subscales, one each for anxiety (HADS-A) and depression (HADS-D). Patients were considered to have risk of anxiety or depression scale score based on a cut-off value of >7 for the respective subscale (30).

### Statistical analysis

Qualitative variables were expressed as relative and absolute frequencies. Parametric variables were expressed as mean and standard deviation (SD) and nonparametric variables as median and 25, 75th percentile (P25, 75). For the comparison of categorical variables, Pearson’s chi-square test was used. For the comparison of numerical variables with a normal distribution, a Student’s t test was used. For the comparison of numerical variables without a normal distribution, a Mann–Whitney U-test was used. To compare more than 2 numerical variables between groups, a Kruskal–Wallis test was used. Univariable logistic regression analysis was performed to identify criteria that were associated with an abnormally low IB or IFR, AMH and SD. A multivariable logistic regression analysis was constructed with the most informative variables in the univariable analysis (*p* < 0.20). A two-tailed *p*-value <0.05 was considered to be significant. We used SPSS statistical package for Windows, version 25.0.

## Results

A total of 163 subjects were enrolled. The groups consisted of 83 men (58 CD), age 28 (24–36) and 25 HC, 31 (26–37) years, and 80 women (50 CD), age 26 (21–36) and 30 HC, age 28 (26–32) years. Table 1 summarizes main demographic characteristics of the overall cohort. Patients and HC had equivalent characteristics in terms of age, BMI or smoking status. Higher education was more frequent among HC than CD patients (84% vs. 47%, *p* = 0.002 and 83% vs. 44*%, p =* 0.003, respectively). On the other hand, a sedentary lifestyle was more frequent among female CD patients compared to HC (46% vs. 3.3%, *p* < 0.001), and were more frequently unemployed vs. HC women (26% vs. 3.3%, *p* < 0.05). The use of anticonceptives was significantly more frequent among HC women compared to CD patients, nevertheless parity and gravidity were not different between both groups. Serum parameters evaluated in CD and HC are shown in Table 2. White blood cell count was significantly higher in both male and female CD patients compared to HC, whereas albumin was lower in CD patients than in HC. Baseline characteristics of the CD patients are shown in Table 3. Up to 24% of women and 8.6% of men had active CD according to the HBI. Perianal disease was present in 16% of women and 22% of men. Eighteen percent of women and 6.9% of men were receiving steroids, 24 and 21% immunosuppressants, and 64 and 69% biologics, respectively.

**Table 1:** Demographic characteristics of Crohn’s patients and controls.

	Male (*n* = 83)			Female (*n* = 80)		
	CD (*n* = 58)	HC (*n* = 25)	*p*-value	CD (*n* = 50)	HC (*n* = 30)	*p*-value
Age, median (IQR), yr	28 (24–36)	31 (26–37)	0.5	26 (21–36)	28 (26–32)	0.4
BMI, median (IQR)	23.7 (22–25.5)	23.2 (22.3–26.2)	0.9	22 (20–25.7)	21.6 (20.6–23.4)	0.8
Smoker status, *n* (%)						
Current	12 (21%)	2 (8.0%)	0.3	13 (26%)	7 (23%)	0.6
Past	6 (10%)	1 (4%)		7 (14%)	2 (7%)	
Non-smoker	40 (69%)	22 (88%)		30 (60%)	21 (70%)	
Regular cannabis consume, *n* (%)	6 (10%)	1 (4%)	0.7	4 (8.0%)	1 (3.3%)	0.3
Regular alcohol consume, *n* (%)	10 (17%)	6 (24%)	0.5	11 (22%)	8 (27%)	0.6
Oral contraceptive use, *n* (%)				19 (38%)	22 (73%)	0.002
Gravidity (*n*, IQR)				2.00 (1.00, 2.00)	3.50 (2.50, 4.25)	0.2
Parity (*n*, IQR)				1.00 (1.00, 2.00)	2.00 (1.75, 2.25)	0.4
Abortions (*n*, IQR)				2.00 (1.00, 2.00)	2.00 (1.00, 3.25)	0.7
Physical exercise, *n* (%)						
sedentary lifestyle	16 (28%)	4 (16%)	0.4	23 (46%)	1 (3.3%)	<0.001
< 3 h/week	18 (31%)	11 (44%)		18 (36%)	13 (43%)	
> 3 h/week	24 (41%)	10 (40%)		9 (18%)	16 (53%)	
Employment status, *n* (%)						
Unemployment	4 (6.9%)	1 (4%)	0.3	13 (26%)	1 (3.3%)	0.017
Student	13 (22%)	3 (12%)		11 (22%)	5 (17%)	
Self-employment	8 (14%)	1 (4%)		3 (6%)	1 (3.3%)	
Employee	32 (55%)	20 (80%)		23 (46%)	23 (77%)	
Retired	1 (1.7%)	0		0	0	
Marital status, *n* (%)						
Single	25 (43%)	4 (16%)	0.057	18 (36%)	16 (53%)	0.4
Married	10 (17%)	5 (20%)		10 (20%)	3 (10%)	
Widowed	0	0		0	0	
Non-marital partner	22 (38%)	16 (64%)		21 (42%)	10 (33%)	
Divorced	1 (1.7%)			1 (2%)	1 (3.3%)	
Education						
No schooling	0 (0%)	0 (0%)	0.002	2 (4.0%)	0 (0%)	0.003
Primary	6 (10%)	2 (8.0%)		4 (8.0%)	0 (0%)	
Secondary	25 (43%)	2 (8.0%)		22 (44%)	5 (17%)	
Higher	27 (47%)	21 (84%)		22 (44%)	25 (83%)	

CD, Crohn’s disease; HC, healthy controls; BMI, body mass index.

**Table 2:** Serum parameters of Crohn’s disease patients and controls.

	Male (*n* = 83)			Female (*n* = 80)		
	CD (*n* = 58)	HC (*n* = 25)	*p*-value	CD (*n* = 50)	HC (*n* = 30)	*p*-value
Mean (± SD)						
CRP, mg/dL	0.49 (0.85)	0.14 (0.28)	0.005	0.5 (1.1)	0.19 (0.18)	0.3
ESR, mm/h	18 (17)	10 (8)	0.042	25 (19)	13 (8)	0.030
White blood cells /uL	7,567 (2348)	5,900 (1549)	0.001	6,862 (2677)	5,628 (1611)	0.030
Hemoglobin, g/dL	15.02 (1.05)	14.85 (0.86)	0.5	12.93 (1.32)	13.34 (0.69)	0.3
Fecal calprotectine, ug/g	148 (149)			174 (223)		
Albumin, g/dL	4.4 (0.3)	4.70 (4.50, 4.80)	0.002	4.1 (0.5)	4.4 (0.4)	0.002
25OH Vitamin D, ng/mL	26.29 (11.93)	21.29 (7.44)	0.2	24.48 (10.26)	31.8 (10.4)	0.001
Vitamin B12, ng/mL	423.8 (142.8)	511.6 (183.8)	0.05	479.12 (262.68)	511.63 (183.9)	0.06

CRP, C-reactive protein; ESR, erythrocyte sedimentation rate.

**Table 3:** Clinical characteristics of Crohn’s disease cohort.

	Male (*n* = 58)	Female (*n* = 50)
Median age at inclusion, (IQR), years	28 (24, 36)	26 (21, 36)
Disease duration, (IQR), years	7.5 (3.0, 13.0)	5.0 (2.0, 10.0)
Active disease at inclusion, *n* (%)	5 (8.6)	12 (24)
Montreal Classification		
Age of onset, *n* (%)		
A1/A2	9 (16)/49 (84)	8 (16)/42 (84)
Location, *n* (%)		
L1/L2/L3/L4	28 (48)/6 (10)/23 (40)/1 (1.7)	21 (42)/13 (26)/15 (30)/1 (2.0)
Behavior, *n* (%)		
B1/B2/B3	41 (71)/8 (14)/9 (16)	39 (78)/6 (12)/5 (10)
Perianal disease, *n* (%)	13 (22)	8 (16)
EIMs, *n* (%)	8 (14)	8 (16)
Intestinal resection, *n* (%)	11 (19)	6 (12)
Treatment		
Current steroids, *n* (%)	4 (6.9)	9 (18)
Current immunosuppressants, *n* (%)	12 (21)	12 (24)
Thiopurines, *n* (%)	11 (18.9)	12 (24)
Methotrexate, *n* (%)	1 (1.7)	
Current biologics, *n* (%)	40 (69)	32 (64)
AntiTNF, *n* (%)	36 (62)	23 (46)
Ustekinumab, *n* (%)	4 (6.8)	9 (18)

EIMs, extraintestinal manifestations.

### Male fertility potential

Serum IB levels were significantly lower in the group of male CD patients compared to HC (177.21 ± 58.96 vs. 234 ± 75.56 pg./mL, *p* = 0.001). When we classified patients by age, these differences were observed in both sub-groups of age, over and under 30 years. IFR was also significantly lower in CD patients than in HC (58.27 ± 59.5 vs. 91.35 ± 60.04, *p* = 0.014); this difference was not observed in the subgroup of men older than 30 years (55.9 ± 52.94 vs. 75.53 ± 42.57, *p* = 0.2).

Results of serum FSH, LH, SHBG, AMH, TT, and FT are shown in Table 4. The prevalence of impaired fertility defined by means IB levels <89 pg./mL or IFR < 23.5, was higher in CD patients (11/57, 19.3%) than in HC (2/24, 8.3%), not reaching statistical significance.

**Table 4:** Sex hormone levels in male Crohn’s disease patients and controls.

	Male (*n* = 83)			Male < 30 years (*n* = 43)			Male > 30 years (*n* = 40)		
Mean (± SD)	CD (*n* = 58)	HC (*n* = 25)	*p*	CD (*n* = 32)	HC (*n* = 11)	*p*	CD (*n* = 26)	HC (*n* = 14)	*p*
IB, pg./mL	177.21 (58.93)	234.12 (75.56)	0.001	175.32 (58.84)	249 (95.33)	0.04	179.46 (60.12)	221.54 (54.73)	0.01
IFR	58.28 (59.26)	91.36 (60.05)	0.014	60.26 (65.40)	110.06 (73.54)	0.04	55.91 (52.94)	75.54 (42.58)	0.25
Low IB (IB < 89 pg./mL), *n* (%)	3 (5.3)	0	0.3	3 (9.7)	0	0.28	0	0	
Low IFR (IFR < 23.5), *n* (%)	11 (19.13)	2 (8.3)	0.3	7 (22.6)	1 (9.1)	0.32	4 (15.4)	1 (7.7)	0.49
IB < 89 ng/mL or IFR < 23.5, *n* (%)	11 (19.13)	2 (8.3)	0.3	7 (22.6)	1 (9.1)	1	4 (15.4)	1 (7.7)	0.49
TT, ng/mL	5.16 (1.52)	6.07 (1.93)	0.055	5.19 (1.6)	6.41 (1.81)	0.042	5.11 (1.45)	5.81 (2.04)	0.21
FT, ng/mL	7.91 (2.06)	6.40	0.2	8.43 (1.98)	7.8 (1.40)	0.2	6.87 (2.06)	6.40	0.85
AMH, ng/mL	5.38 (2.56)	7.91 (4.20)	0.005	5.78 (2.4)	8.01 (4.38)	0.04	4.88 (2.69)	7.8 (4.2)	0.01
FSH, IU/L	4.27 (3.16)	3.47 (1.76)	0.3	4.23 (3.65)	3.38 (2.19)	0.47	4.32 (2.52)	3.54 (1.41)	0.28
LH, IU/L	6.01 (2.56)	4.87 (1.96)	0.10	6.17 (2.96)	5.12 (2.05)	0.28	5.81 (1.99)	4.68 (1.95)	0.09
SHBG, nmol/L	29.20 (9.75)	41.16 (14.43)	<0.001	27.92 (9.59)	43 (15.52)	0.0001	30.77 (9.9)	39.71 (13.92)	0.02

IB, Inhibin B; IFR, Inhibin/FSH ratio; TT, Total testosterone; FT, Free testosterone; AMH, Antimüllerian hormone; FSH, follicle-stimulating hormone; LH, luteinizing hormone; SHBG, Sex hormone binding globulin.

Regarding male hormone profile, TT, SHBG, and AMH levels were significantly lower in male CD patients compared to HC, whereas calculated FT showed no differences between both groups, regardless of age.

### Ovarian reserve

**Figure 1:**
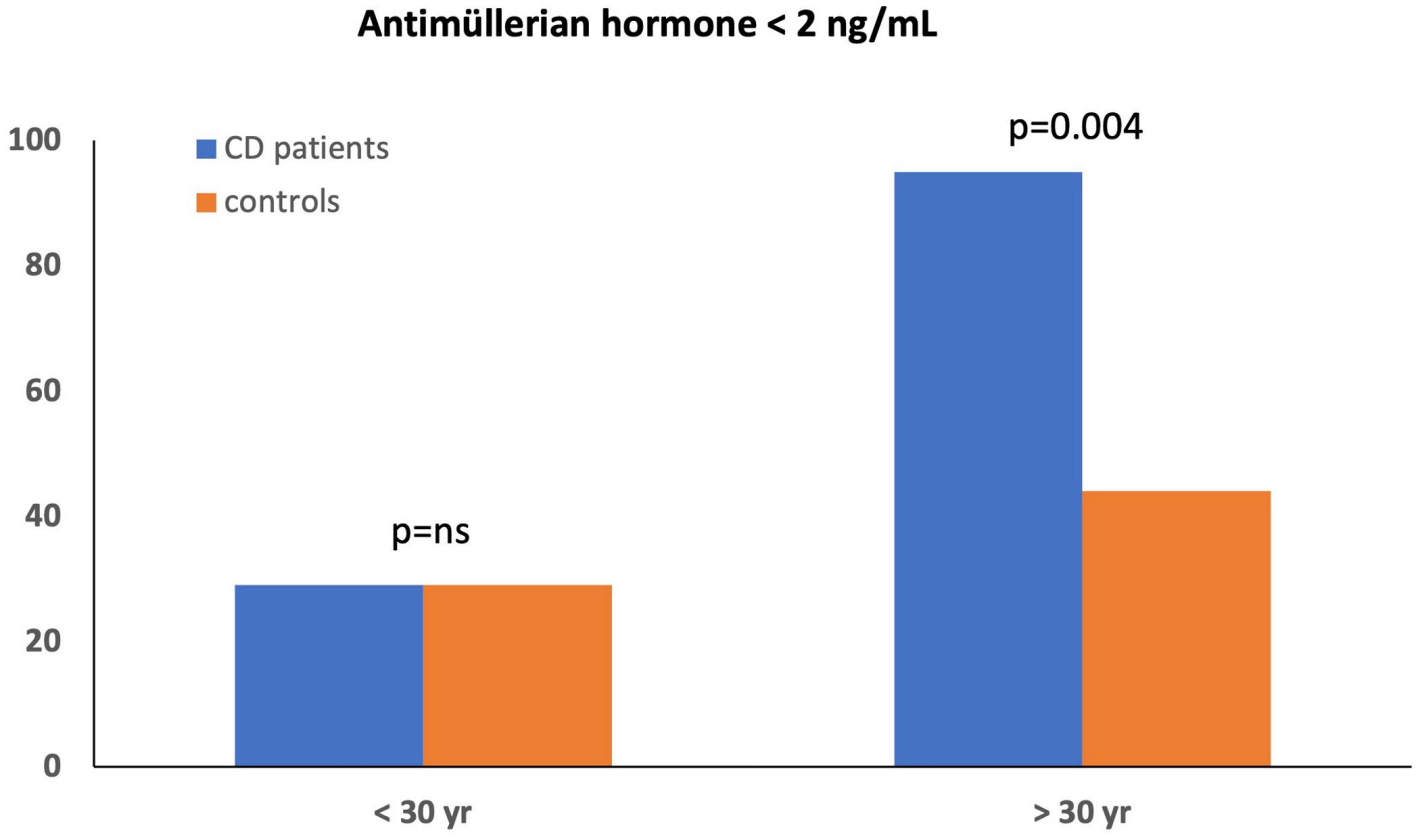
AMH levels distributed by age in female CD patients and controls.

Table 5 summarizes sex hormone levels in the women cohort. Serum AMH levels were similar in female CD patients and HC (2.56 ± 2.03 vs. 2.97 ± 2.03 ng/mL, *p* = 0.4). The rate of low serum AMH levels according to age using local laboratory cut-offs did not differ between groups (11/50, 22% in female CD vs. 7/30, 23% in HC, *p* = 0.78). When we divided women by age (<30 and > 30 years), the serum AMH levels remained comparable between CD and HC only in the subgroup <30 years (3.49 ± 2.07 vs. 3.39 ± 2.09 ng/mL, *p* = 0.9). However, AMH levels among CD women >30 years were significantly lower compared to HC (1.16 ± 0.76 vs. 2.14 ± 1.68 ng/mL, *p* = 0.033). In addition, significantly higher proportion of CD women over 30 years displayed a low ovarian reserve (<2 ng/mL) compared to HC (18/19, 90% vs. 4/9, 40%, *p* = 0.004) (Figure 1). In a secondary analysis of reduced ovarian reserve defined as AMH ≤1.1 ng/mL, the proportion of patients with low ovarian reserve did not differ between groups.

**Table 5:** Sex hormone levels in female Crohn’s disease patients and controls.

Mean (± SD)	CD female (*n* = 50)	HC (*n* = 30)	*p*-value	CD female <30 years (*n* = 31)	HC < 30 years (*n* = 21)	*p*-value	CD female >30 years (*n* = 19)	HC > 30 years (*n* = 9)	*p*-value
AMH, ng/mL	2.56 (2.03)	2.97 (2.03)	0.39	3.49 (2.07)	3.39 (2.09)	0.78	1.15 (0.74)	2.13 (1.68)	0.03
Low AMH*, *n* (%)	11 (22)	7 (23.3)	0.9	7 (23.3)	4 (20)	0.78	4 (20)	3 (30)	0.8
AMH < 2 ng/mL, *n* (%)	27 (54)	10 (33.3)	0.073	9 (30)	6 (30)	0.9	18 (90)	4 (40)	0.004
AMH <1.1 ng/mL, *n* (%)	13 (26)	9 (30)	0.68	2 (6.7)	4 (20)	0.15	11 (55)	5 (50)	0.79
FSH, IU/L	7.04 (3.44)	7.37 (4.62)	0.71	6.12 (1.92)	6.03 (2.61)	0.88	8.37 (4.62)	10.05 (6.49)	0.42
LH, IU/L	8.19 (4.39)	6.65 (3.61)	0.2	8.16 (4.35)	6.30 (3.38)	0.11	8.22 (4.55)	7.35 (4.13)	0.61
Estradiol, pg./mL	62.79 (69.19)	45.68 (56.74)	0.25	60.54 (60.83)	36.32 (23.02)	0.09	66.17 (81.73)	64.41 (93.11)	0.95

AMH, Antimüllerian hormone; CD, Crohn’s disease; HC, healthy controls; FSH, follicle-stimulating hormone; LH, luteinizing hormone. *Low AMH according to our laboratory hospital cut-offs.

Mean serum levels of LH, FSH, and estradiol were not different in CD women compared to HC.

### Factors associated with impaired fertility potential in CD patients

**Figure 2:**
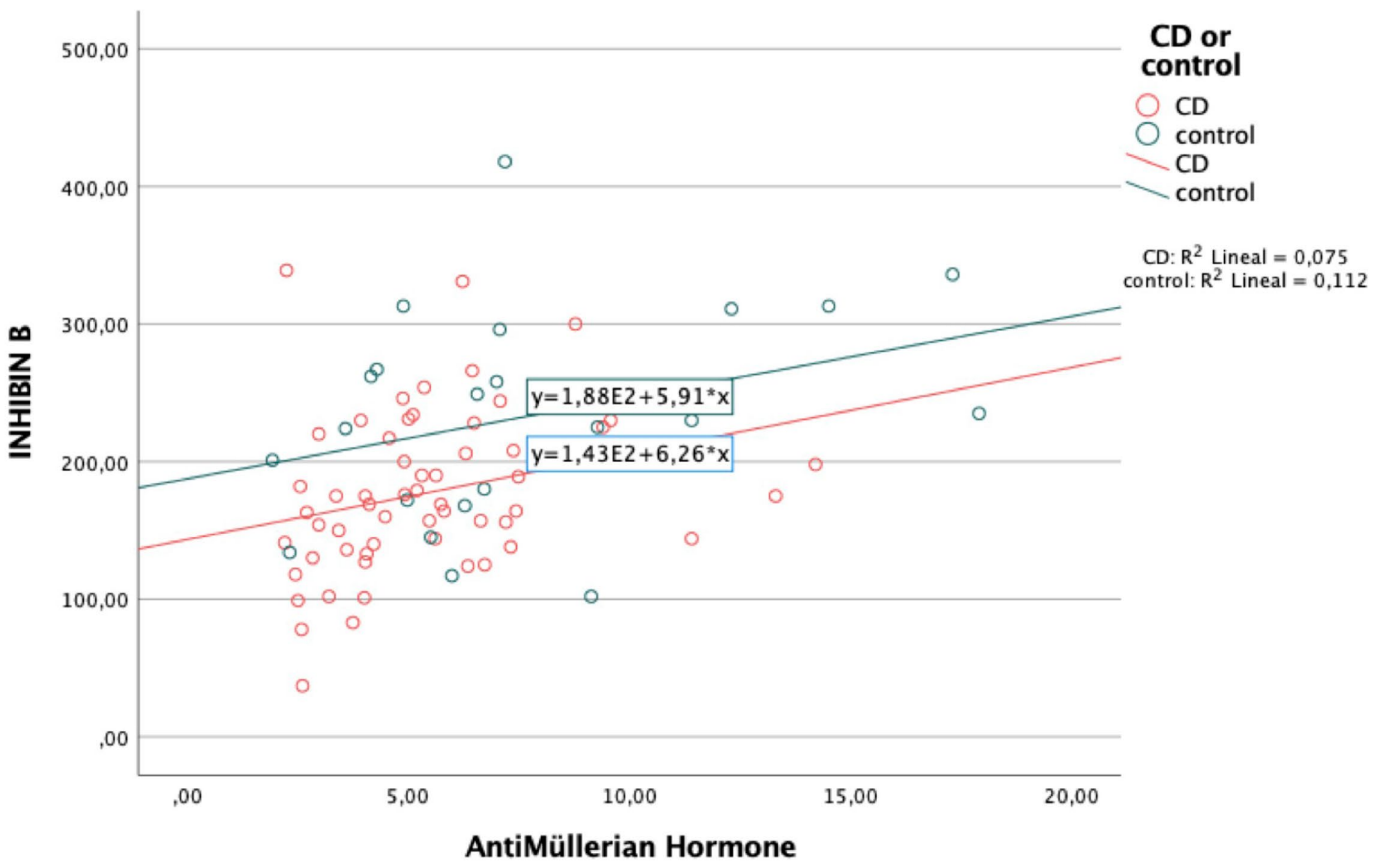
Correlation between Inhibin B and AntiMüllerian hormone levels in men.

Having low levels of IB in male CD patients were only associated with having low levels of AMH (*r* = 0.075, *p* = 0.01) (Figure 2). No association between IB levels and any of the other parameters related to CD was detected.

Age above 30 years and treatment with immunosuppressants were identified as risk factors associated with low ovarian reserve in CD women (AMH < 2 ng/mL) in the univariable analysis (Table 6). The multivariable analysis showed that age > 30 years was the single independent predictor associated with low ovarian reserve, OR:17.24, CI 95% (2.80–106.09), *p* = 0.002.

**Table 6:** Univariable and multivariable analysis of factors associated with low ovarian reserve (AMH ≤ 2 ng/mL) in Crohn’s disease female patients.

			Univariable analysis		Multivariable analysis	
	AMH < 2 ng/mL (*n* = 27)	AMH > 2 ng/mL (*n* = 23)	OR (95%CI)	*p*-value	OR (95%CI)	*p*-value
Age > 30 years, *n* (%)	18 (66.7)	2 (8.7)	21 (4–110.05)	0.0001	17.24 (2.80–106.09)	0.002
Mean duration of the disease >5 years, *n* (%)	17 (63)	9 (39.1)	2.64 (0.84–8.31)	0.09		
Smokers, *n* (%)	10 (37)	3 (13)	3.92 (0.92–16.6)	0.063		
Previous intestinal resection, *n* (%)	3 (11.1)		0.83 (0.15–4.59)	0.83		
EIMs, *n* (%)	6 (22.2%)	2 (8.7)	3 (0.54–16.6)	0.19		
Perianal disease, *n* (%)	6 (22.2%)	2 (8.7)	3.14 (0.40–24.59)	0.19		
Location of the disease, *n* (%)			0.6 (0.27–1.42)	0.83		
Ileal (L1)	11 (40.7%)	10 (43.5)				
Colonic (L2)	7 (25.9)	6 (26.1)				
Ileocolonic (L3)	8 (29.6)	7 (30.4)				
Upper gastrointestinal tract (L4)	1 (3.7)					
Behavior of the disease, *n* (%)			3.3 (0.68–15.89)	0.25		
Nonstricturing, non penetrating (B1)	23 (85.2)	16 (69.6)				
Stricturing (B2)	3 (11.1)	3 (13)				
Penetrating (B3)	1 (3.7)	4 (17.4)				
Active disease, *n* (%)	6 (22.2%)	6 (26.1)	0.81 (0.22–2-97)	0.75		
Current steroids, *n* (%)	5 (18.5)	4 (17.4)	1.08 (0.25–4.60)	0.91		
Current immunosuppressants, *n* (%)	10 (37)	2 (8.7)	6.17 (1.1–32)	0.03		
Current biologic therapy, *n* (%)	16 (59.35)	16 (69.6)	1.79 (0.31–10.30)	0.44		

AMH, antiMüllerian hormone; OR, odds ratio; CI, confidence interval; EIMs, extraintestinal manifestations.

### Sexual dysfunction (SD) and psychologic functioning

Supplementary Tables S1, S2 summarize SD and psychologic functioning in CD patients and HC. Up to 24%(14/58) of male patients and 4% (1/25) of controls showed SD measured by an abnormal IIFE score (*p* = 0.031). Moreover, IIFE values scores differed between male patients and controls, as well as overall satisfaction domain, with no differences in the analysis of the remaining domains (Supplementary Table S1). Up to 15% of patients had erectyl disfunction but none among controls (*p* = 0.08).

Female SD, measured by an abnormal FSFI score, was more frequent among patients [19/50 (38%)] than controls [3/30 (10%), *p* = 0.017]. FSFI scores were significantly lower in patients compared to controls in the desire and lubrication domains (Supplementary Table S2).

We found no association between SD and fertility potential assessed by IB level < 89 pg./mL or IFR < 23.5 (28.6% vs. 16.3%, *p* = 0.31) or low ovarian reserve, using a cut-off of AMH < 2 ng/mL (68% vs. 46.7%, *p* = 0.13).

Multivariable regression model showed that anxiety was the only independent risk factor for SD in women with CD [OR: 4.48; 95%CI (1.08–18.4, *p* = 0.38)].

## Discussion

There is a lack of agreement on the impact of CD on fertility. In this study we show that IB levels and IFR are lower in male CD patients, regardless of age, and that AMH levels are decreased in women with CD older than 30 years, compared to age-matched healthy controls. These results suggest that CD may have an intrinsic impact on spermatogenesis and ovarian reserve and thus on future fertility potential.

The prevalence of impaired fertility potential among men diagnosed with CD was 19% in our series. This prevalence was numerically higher compared to HC, not reaching statistical significance. Other studies reported a prevalence of 14–15% for non-selected patients under 45 years (7). Moreover, we found that male patients had significant lower IB levels and IFR compared to controls. There is a paucity of data on IB or IFR values in patients with IBD, particularly in men diagnosed with CD. Nevertheless, a study designed to assess whether phthalates alter serum hormones in male patients diagnosed with IBD under mesalamine reported baseline IB levels similar than ours (31). They concluded that the exposure to DBP during long periods of time disrupts gonadal hormones among IBD men. However, in our series we did not find correlation among any IBD treatment and IB levels, including mesalamine. It could be related to the small number of CD patients treated with mesalamine in our study, due to clinical guidelines recommendations against its use for maintenance of medically induced remission in patients with CD (32).

It has been reported that chronic inflammation plays a significant role in the development of Sertoli cell dysfunction. This conclusion is drawn from the observed impact of the proinflammatory cytokine IL-1 on IB secretion, as evidenced by lower IB levels in Sertoli cell cultures when IL-1 is introduced (33). Contrary to this concept, levels of IB were not affected by the inflammatory biomarkers, CRP and fecal calprotectin, or by CD clinical activity in our series. This fact could be related with the high percentage of CD patients that were in remission in our study.

Other factors have been associated to impaired male fertility potential such as age and exercise. In fact, inverse relationships between age and IB and IFR have been reported (8, 34), and these associations are stronger in subjects older than 30 years. Interestingly, our results show that IB levels were significantly lower comparing to HC in patients either younger or older than 30 years. Moreover, IRF was also lower in CD patients compared to controls but only for the subgroup of patients under 30 years. These findings could reflect that CD itself causes this decrease in IB levels even in younger patients. Regarding physical activity, negative impact of prolonged exercise on IB levels have been reported in the general population (35). The regression analysis performed in our study showed that physical exercise was not related to IB levels or IFR, probably because the physical exercise intensity of the participants was below the threshold associated with a deleterious effect on IB levels.

Regarding risk factors for low IB levels, only AMH levels were associated to IB levels in our CD male cohort. AMH is a member of the transforming growth factor (TGF) beta superfamily, produced by the Sertoli cells (36) and plays a key role in the regression of the Mullerian ducts during male sex determination in the fetus. There is limited evidence for a role of AMH in spermatogenesis but lower levels of AMH in serum have been reported in subfertile compared to normal men (37, 38). In accordance with these data our study found lower AMH in CD patients, this finding alongside lower IB and IFR suggests an impaired Sertoli cell function in our CD cohort, particularly in young male patients under 30 years, the implications of which should be stated in further studies.

Regarding female fertility, it remains controversial whether CD impairs ovarian reserve. AMH is a reliable, indirect marker of functional ovarian reserve that can be useful to predict a poorer ovarian reserve or a weaker response to ovarian stimulation (39). We found that serum AMH levels were similar in CD women compared to HC in the whole female cohort. In accordance with our data, Koller et al. reported no difference in AMH levels in a case control study, including only CD patients in clinical remission (16). In our series 75% of CD women were also clinically inactive. On the contrary, other studies yielded that CD women have lower AMH levels in CD vs. control females, matched by age and other demographic features, although the percentage of active patients in these studies was above 40% (14). Therefore, it can be speculated that the lack of difference in AMH levels between both groups could be due to the mainly quiescent CD population of our study. However, when we divided patients over or under 30 years, we found that AMH levels were significantly lower in CD women versus controls, underscoring that the risk of low ovarian reserve was higher among CD women compared to general population in this age subgroup.

There is no complete consensus on normal AMH values, because different studies use different cut-offs to define low ovarian reserve (27, 40, 41). We adopted the threshold for low ovarian reserve as AMH < 2 ng/mL, according to previous studies that have focused on ovarian reserve in women with CD (11, 14), finding that up to 90% of CD women older than 30 years had low ovarian reserve, while it happens in only 40% of HC over 30 years (*p* = 0.004). AMH levels are negatively correlate with age in adult women (42). In fact, the regression analysis that we performed found that age was the single independent factor predicting low ovarian reserve (AMH < 2 ng/mL) [OR = 17.24, CI 95% (2.80–106.09, *p* = 0.002)]. Not only age but also several other factors can alter ovarian reserve in CD women such as disease location, duration or smoking habit. Freouer (11) described that colonic location was associated to altered ovarian reserve suggesting that the proximity between the colon and the pelvis may result in a higher level of chronic inflammation than in other disease locations. The harmful impacts of tobacco on ovarian function have been a subject of discussion for a long time (43). Nevertheless, smoking habit, CD location and disease duration (greater or less than 5 years) were not related to ovarian reserve in our study. One possible explanation could be that our CD population did not smoke more frequently than controls. In our study we only determined AMH as a marker of ovarian reserve without measuring the number of follicles. In fact, most studies investigating AMH levels in women with IBD do not include antral follicle counting, given the high reliability of AMH as a marker of ovarian reserve. On the other hand, Luteinizing hormone (LH) and follicle-stimulating hormone (FSH) play complementary roles in follicle development and ovulation via a complex interaction in the hypothalamus, anterior pituitary gland, reproductive organs, and oocytes. Impairment of the production or action of gonadotropins causes relative or absolute LH and FSH deficiency that compromises gametogenesis and gonadal steroid production, thereby reducing fertility (44). However, we did not find different levels of LH, FSH and estradiol between women with CD and HC, even when women were stratified by age.

Sexual dysfunction is a multifactorial, progressive and age-related problem (45, 46). It has been described that women with subfertility have greater prevalence of SD. We included SD assessment in our study, finding that this was significantly more frequent among male (24% vs. 4%, *p* = 0.031) and female (38 vs. 10%, *p* = 0.017) CD patients than in controls, as previously described (18, 47). Anxiety was the only factor associated to SD among women with CD. This result is consistent with previous reports that support that psychological factors such as anxiety, depression or fatigue are determinant for SD in IBD (48). Studies on the association between female subfertility and SD have reported conflicting results. Some of them found no significant difference in the prevalence of SD between subfertile and fertile women similar to the present study findings (49). On the other hand, hypogonadism, diagnosed by the presence of hypogonadal symptoms and low serum testosterone levels, is linked to infertility and SD. With age, there is a decrease in testosterone and an increase in SHBG in men, which can result in decreases in bone density, muscle strength, and libido and in erectly dysfunction (50). In the current study we found lower total testosterone levels in CD patients <30 years compared to controls, but no correlation could be demonstrated between male sexual SD and any sex hormone levels, taking into account that the study was not powered to, specifically, evaluate risk factors for SD because it was a secondary endpoint.

Our study had several important strengths. It simultaneously evaluates both male and female fertility using a cross-sectional design. In addition to studying IB, IFR and AMH, that are reproducible and reliable biomarkers of Sertoli cell function, ovarian reserve and fertility potential, we performed an added comprehensive panel of sex hormones in all patients, thereby enhancing the value of the obtained results. Furthermore, patients and controls were matched for age, and were comparable regarding BMI and smoking habit, mitigating biases associated with these key factors associated to fertility. Finally, SD was included as secondary endpoint and its assessment excluded the influence on the fertility potential of the patients. Nevertheless, it must be considered that most patients in our series were quiescent, and the results may not be generalized to active patients. Also to be considered, a 2 ng/mL threshold was used to define low ovarian reserve, so lower thresholds may yield different results.

In conclusion, we have found that the IB levels and IRF are lower in male CD patients, as marker of impaired Sertoli cell function, compared to healthy controls regardless of age. This finding should be confirmed in further prospective studies to set up its potential impact on spermatogenesis. We also demonstrated that AMH levels are significantly decreased in CD women older than 30 years, reinforcing the key role of age on ovarian reserve, particularly for patients with CD even in remission. These findings could be helpful to guide CD patients with desire for offspring.

## Data availability statement

The original contributions presented in the study are included in the article/Supplementary material, further inquiries can be directed to the corresponding author.

## Ethics statement

The studies involving humans were approved by Research Ethics Committee of the Hospital Universitario Dr Balmis, Alicante, Spain (PI2019/093). The studies were conducted in accordance with the local legislation and institutional requirements. The participants provided their written informed consent to participate in this study.

## Author contributions

AG: Conceptualization, Data curation, Formal analysis, Funding acquisition, Investigation, Methodology, Project administration, Resources, Software, Supervision, Validation, Visualization, Writing – original draft, Writing – review & editing. RM-P: Conceptualization, Data curation, Investigation, Methodology, Project administration, Writing – original draft, Writing – review & editing, Formal analysis, Funding acquisition, Resources, Software, Supervision, Validation, Visualization. PZ: Conceptualization, Data curation, Formal analysis, Methodology, Project administration, Software, Supervision, Validation, Writing – review & editing. CM: Investigation, Methodology, Writing – review & editing. AR: Conceptualization, Data curation, Investigation, Writing – review & editing. LS-R: Conceptualization, Data curation, Supervision, Writing – review & editing. MT: Investigation, Methodology, Writing – review & editing. RA: Investigation, Methodology, Writing – review & editing. VM-T: Data curation, Investigation, Software, Writing – review & editing, Project administration. LB: Data curation, Investigation, Software, Writing – review & editing. OB-G: Data curation, Investigation, Validation, Writing – review & editing. JC: Conceptualization, Data curation, Investigation, Methodology, Supervision, Writing – review & editing. PS: Investigation, Writing – review & editing, Data curation. BH: Conceptualization, Data curation, Investigation, Methodology, Supervision, Writing – review & editing. PB: Conceptualization, Methodology, Writing – review & editing. OM-P: Conceptualization, Methodology, Supervision, Visualization, Writing – review & editing. LM-V: Conceptualization, Data curation, Investigation, Methodology, Supervision, Writing – original draft, Writing – review & editing.
