# Supplementary material for: Inhibin B and antiMüllerian hormone as surrogate markers of fertility in male and female Crohn’s disease patients: a case-control study
Source: Front Med (Lausanne). 2024 Apr 25;11:1374603. doi: 10.3389/fmed.2024.1374603 (PMC11080652; doi:10.3389/fmed.2024.1374603)
Supplement: Supplementary file 1 [file Table_1.docx]

## **Supplementary Table 1. Sexual dysfunction and psychologic functioning in male CD patients and controls**

|  | Male (n=83) | |  |
| --- | --- | --- | --- |
|  | CD (n=58) | HC (n=25) | p |
| IIEF, median (IQR) | 65 (45, 70) | 69 (66, 72) | 0.013 |
| Intercourse satisfaction domain, median (IQR) | 13.00 (12.00, 15.00) | 14.00 (12.75, 15.00) | 0.2 |
| Orgasmic function domain, median (IQR) | 10.00 (10.00, 10.00) | 10.00 (10.00, 10.00) | 0.3 |
| Sexual desire domain, median (IQR) | 7.00 (6.00, 9.00) | 8.00 (7.00, 9.00) | 0.12 |
| Overall satisfaction domain, median (IQR) | 8.00 (6.00, 9.00) | 9.00 (8.00, 10.00) | 0.028 |
| Erectyl function domain, median (IQR) | 30.00 (28.00, 30.00) | 30.00 (29.00, 30.00) | 0.6 |
| Male SD (IIEF < 42.9) n(%) | 14 (24) | 1 (4%) | 0.031 |
| Erectyl dysfunction (IIEF erectyl function < 26) n(%) | 7 (15) | 0 (0) | 0.087 |
| HADS (Anxiety) median (IQR) | 5.0 (3.0, 8.0) | 4.0 (1.0, 6.0) | 0.042 |
| HADS-Anxiety >7, n(%) | 16 (28%) | 4 (16%) | 0.3 |
| HADS-Depression median (IQR) | 2.00 (1.00, 5.00) | 1.00 (1.00, 3.00) | 0.3 |
| HADS-Depression >7, n(%) | 7 (12%) | 3 (12%) | 0.9 |

Values are number of subjects and (percentage) unless otherwise specified IIEF: International Index for Erectile Function. Abnormal erectile function: defined as < 26, Abnormal IIFE: defined as <42.9, IQR:interquartile ratio, SD:sexual dysfunction, HADS: Hospital Anxiety and Depression Scale.
