# Supplementary material for: Inhibin B and antiMüllerian hormone as surrogate markers of fertility in male and female Crohn’s disease patients: a case-control study
Source: Front Med (Lausanne). 2024 Apr 25;11:1374603. doi: 10.3389/fmed.2024.1374603 (PMC11080652; doi:10.3389/fmed.2024.1374603)
Supplement: Supplementary file 2 [file Table_2.docx]

**Supplementary Table 2. Sexual dysfunction and psychological functioning in female Crohn´s disease patients and controls**

|  | Female (n=80) | |  |
| --- | --- | --- | --- |
|  | CD (n=50) | HC (n=30) | p |
| FSFI, median (IQR) | 28.0 (24.3, 31.8) | 30.5 (29.2, 33.5) | 0.006 |
| Desire domain, median (IQR) | 5.00 (4.00, 7.00) | 7.00 (5.00, 7.00) | 0.008 |
| Arousal domain, median (IQR) | 9.0 (6.0, 11.0) | 11.0 (9.0, 12.0) | 0.069 |
| Lubrication domain, median (IQR) | 10.0 (7.0, 13.0) | 13.0 (9.2, 14.0) | 0.044 |
| Orgasm domain, median (IQR) | 4.80 (4.00, 5.60) | 5.20 (4.40, 6.00) | 0.13 |
| Global satisfaction domain, median (IQR) | 5.20 (4.00, 6.00) | 5.20 (4.35, 6.00) | 0.6 |
| Pain domain, median (IQR) | 4.80 (3.60, 6.00) | 5.60 (4.80, 6.00) | 0.2 |
| Female SD (FSFI < 26.55) n(%) | 19 (39%) | 3 (12%) | 0.017 |
| HADS (Anxiety) median (IQR) | 7.5 (5.0, 9.8) | 5.0 (3.0, 7.0) | 0.015 |
| HADS-Anxiety >7, n(%) | 25 (50%) | 6 (21%) | 0.013 |
| HADS-Depression median (IQR) | 4.00 (1.00, 5.00) | 1.00 (1.00, 2.75) | <0.001 |
| HADS-Depression >7, n(%) | 6 (12%) | 0 (0%) | 0.082 |

Values are number of subjects and (percentage) unless otherwise specified FSFI: Female Sexual Function Index. Abnormal FSFI: defined as < 26.5, HADS: hospital anxiety and depression scale, IQR: interquartile range.
